# Current Issues in Social Medicine and Public Health from the Viewpoint of Early-career Researchers: Summary of Opinions at the Social Medicine Young Retreat

**DOI:** 10.31662/jmaj.2021-0219

**Published:** 2022-06-17

**Authors:** Kouji H. Harada, Koji Hara, Takuma Yamamoto, Motoki Endo, Mitsuo Uchida, Keisuke Kuwahara

**Affiliations:** 1 Department of Health and Environmental Sciences, Kyoto University Graduate School of Medicine, Kyoto, Japan; 2 Department of Healthcare Economics and Quality Management, Kyoto University Graduate School of Medicine, Kyoto, Japan; 3 School of Economics and Business Administration, Yokohama City University, Kanagawa, Japan; 4 Department of Legal Medicine, Hyogo Medical University, Hyogo, Japan; 5 Department of Public Health, Juntendo University Faculty of Medicine, Tokyo, Japan; 6 Department of Public Health, Graduate School of Medicine, Gunma University, Gunma, Japan; 7 Teikyo University Graduate School of Public Health, Tokyo, Japan

**Keywords:** early-career, researchers, professionals, challenges, social medicine, public health

## Abstract

The coronavirus disease 2019 (COVID-19) pandemic highlighted the importance of research, practice, social contribution, and education in social medicine and public health, which relate to the core mission of universities. Early-career researchers and professionals play an important role in these domains, but little is known about the challenges and issues they encountered or recognized during this pandemic. Therefore, we summarized the opinions of 37 participants (30 early-career researchers and seven senior researchers) on this issue from discussions at the Social Medicine Young Retreat, 2019, of the Japanese Medical Science Federation. The retreat was initially planned to be held during March 5-6, 2020 in Yamanashi but was changed to be held virtually on March 5, 2021. Early-career researchers participated in group discussions on how social medicine should transform itself to serve the public during the COVID-19 pandemic. Afterward, each group provided opinions on challenges and issues in social medicine. For example, participants perceived difficulties in implementing research in a timely way and the lack of multidisciplinary collaboration. They recognized challenges in continuing practice because of the limited evidence on COVID-19. On social contribution, they described difficulties in communicating risk as professionals. They also noted issues arising from online teaching and learning. One group suggested that the essence of social medicine did not need to be changed, but methodologies should be updated to tackle multiple existing challenges. These opinions may not cover all issues but could help establish a better relationship between medicine and society in a bottom-up manner. The continuous promotion of interdisciplinary collaboration in social medicine (and basic and clinical medicine) would provide ideas to solve these issues at scale. Organizational support is warranted to ensure sustainability and scalability of these actions.

## Background

The coronavirus disease 2019 (COVID-19) pandemic highlighted the importance of research, practice, social contribution, and education in the fields of social medicine and public health. Early-career researchers and professionals play an important role in these domains, but their experiences and perceptions during this pandemic are not well understood. Therefore, this paper summarizes the challenges and issues they encountered or recognized during the COVID-19 pandemic about how to enhance the relationship between society and medicine. The opinions in this article are based on discussions during the Social Medicine Young Retreat, held in March 2021, hosted by the Japanese Medical Science Federation (JMSF).

### The Japanese Medical Science Federation

The JMSF is the sole and comprehensive Japanese association for academic societies in medicine, representing the field of medical science in Japan. It grew out of the Japanese Association of Medical Sciences, which was established in 1902 ^(1)^. In February 2022, the JMSF comprised 138 academic societies as members (104 societies in clinical medicine, 19 in social medicine, and 15 in basic medicine). The JMSF has played an important cross-cutting role in the promotion of medical science and technology among its member medical societies. For example, it published an expert opinion paper on COVID-19 that summarized recommendations by many academic societies to address public needs ^(2)^.

### The context for the Social Medicine Young Retreat

Networking is important for both research ^(3)^ and career progression ^(4)^ among early-career researchers. To promote young researchers belonging to societies in the division of basic medicine of the JMSF, the Young Retreat for Basic Medicine was held in March 2019 (https://www.jbsoc.or.jp/seika/wp-content/uploads/2018/11/jmsfnews20181113.pdf). However, opportunities for building networks at the national level are limited for social medicine in Japan. Therefore, the JMSF initiated a nationwide cross-disciplinary platform across 19 societies to create a Social Medicine Young Retreat that would promote early-career researchers and enhance their career development in social medicine. The aim of this retreat was to promote mutual exchange among young members belonging to the 19 member societies (Table S1) in the social division of the JMSF.

## Outline of the Retreat

To organize the retreat for social medicine, an executive committee was formed in May 2019. Eleven early-career researchers from 10 member societies were nominated as committee members by the member societies. Two directors from the JMSF were in charge of the retreat and one senior researcher assisted with its management. The JMSF provided financial and administrative support. After active discussions among committee members, the theme of the retreat was set as “Creating the Future of Medicine and Society.” The retreat was designed to promote multidisciplinary communication, networking, and research collaboration between member societies.

The site for the Social Medicine Young Retreat was determined by considering the history of medicine in Japan. The executive committee selected Fuefuki city in Kofu Basin, Yamanashi Prefecture, because it is where the pathology of schistosomiasis japonica, a severe endemic disease, was clarified and the disease was finally eradicated in Japan by efforts in basic, clinical, and social medicine ^(5)^. Japan is now considered to be the first country in the world to have eradicated schistosomiasis ^(5)^.

Each member society in the social division of the JMSF was asked to nominate up to three participants. The criteria for participation were being an early-career researcher (generally aged up to 45 years and ranging from graduate students to professor positions) belonging to member societies in the social division of the JMSF and having an interest in research collaboration. A special website was launched for the announcement (Figure S1).

The retreat was originally planned to be held on March 5 and 6, 2020 (Thursday-Friday). However, as a result of the COVID-19 pandemic, a decision was made in February 2020 that it should be postponed. As there was no sign of the pandemic’s end, the executive committee hosted a shorter retreat on Friday, March 5, 2021, changing the style from in-person to online (Table S2). In total, 37 researchers (30 early-career researchers [primarily faculty members] from the 15 member societies and seven senior researchers, including one observer, one senior committee member, and five directors of the JMSF) joined the online retreat.

After an opening ceremony, the participants introduced themselves. The early-career researchers then participated in group work. The participants were divided into six groups, each including four to five people, with a balance of disciplines or societies. Over two sessions, participants exchanged opinions on the group discussion topic: “How should social medicine transform itself for the public under the COVID-19 pandemic?” A cloud-based slide-sharing and editing service was used during the group work as an advantage of an online meeting. After group discussions in these two sessions, each group presented a summary of their views. Senior researchers provided brief comments on the early-career researchers’ opinions in the final session.

## Issues Raised during the Discussion

Drawing on the participants’ opinions, we summarized the challenges or issues that early-career participants had encountered in social medicine. We classified them into four categories: research, practice, social contribution, and education, which relate to the core mission of universities (Table 1). We set the opinions in the context of available evidence on these issues, especially in Japan, to gain a better understanding of the situation. Relevant evidence is provided in the Supplementary text.

**Table 1.** Examples of Issues Raised by Participants through Discussions at the Social Medicine Young Retreat, 2019.

Main theme	Subtheme
Research	Timely research, research environment, network, data insufficiency, research topic, and publishing (e.g., use of preprints)
Practice	Implementing infection control measures even without sufficient evidence, multidisciplinary collaboration, information literacy because of advances in online communication, health management for people working from home, and flexible modification and operations amid the crisis
Social contribution	Dialogue with society, measures against infodemics, science and risk communication, health literacy in civil society, and outreach activities
Education	Issues related to online classes: skills and effects of online classes on health

### Research

Participants identified the following challenges in this research:

(1) Establishing a research system for the COVID-19 pandemic and providing timely evidence when research activities were restricted;

(2) Communication with stakeholders (from the preparedness phase);

(3) Building a network that links diverse fields of expertise;

(4) Lack of data (e.g., on vulnerable populations, including socioeconomically disadvantaged people, and balance between economy and public health); and

(5) Use of preprints for the timely sharing of findings.

Related issues have previously been summarized by other academics in Japan (e.g., by the Science Council of Japan, the representative organization of the Japanese scientist community for all fields of sciences). Participants did not mention a negative impact of COVID-19 on their research motivation, but another Japanese study showed that COVID-19-related restrictions were associated with a decrease in research motivation and anxiety about future research activities, especially among young researchers.

There may be other pandemics and health emergencies in the future. Therefore, participants suggested that it would be helpful to examine the effectiveness of current policies on COVID-19 using various data to prepare for future crises. To promote research, participants recognized the importance of sharing data collected by public-sector organizations and building a system to share accurate data in a timely way. Similar proposals for Japan were previously documented in detail by the Science Council of Japan. To promote research on the impact of health-related policies, sharing data on policies is important. However, to our knowledge, there is no database to support this in Japan.

### Practice

In terms of practice, the participants mentioned the challenges in implementing facility-level infection prevention and control measures (e.g., in workplaces, prisons, and other public spaces). From the viewpoint of legal medicine, one group shared particular challenges in infection control related to forensic autopsies, which need a strong focus on infection control even without a pandemic. Across society, there may be considerable inequities in these challenges. An example is a disparity in the implementation of infection control by company size in occupational settings.

Participants described challenges related to the necessity to respond to emergencies without sufficient evidence during the pandemic. They perceived difficulties in solving the practical issues or questions related to COVID-19 (e.g., health management for individuals working from home, effective vaccination procedure in remote settings). They had also encountered challenges in cooperating with experts in other specialties during the pandemic.

Another point of difficulty is that guidelines on implementing practice during the pandemic have been published and updated ^(2)^, but adaptation of these guidelines to each setting needs advanced skills and decision making. Therefore, evidence on how to implement practice is needed. Some participants mentioned that there is a great deal of information on COVID-19, which some practitioners were finding hard to digest. Participants suggested that it might be helpful to discuss the role of the information ecosystem, including mass media and social networking services, to improve the response to future crises.

Some participants talked about disruptions in healthcare among individuals living in the community (e.g., reductions in hospital or clinic visits among patients with cancer from remote islands). Participants also raised concerns about the negative impacts on healthcare use of the economic disruption from the pandemic, especially among individuals involved in precarious work or those receiving low incomes. However, studies in Japan are still limited.

Participants were also concerned about physician shortages in local areas, especially where that led to suspension of healthcare services. To maintain the quality of medical services, it may be helpful to use information and communication technology, especially in rural areas. Physicians’ working hours are limited by law in Japan; therefore, multi-sectoral discussion is warranted to ensure that essential healthcare services are not disrupted.

Various types of online and digital transformations have advanced during this pandemic. Participants reiterated the importance of ensuring information technology (IT) literacy among healthcare stakeholders (e.g., physicians, nurses, other health professionals, patients, administrators, and organizational managers). This perception may be partly because of the experience of trouble using digital technologies. Disparities in telemedicine use had widened across generations during the pandemic. Therefore, a generation-specific approach may be needed to improve IT literacy.

In relation to digital transformation, participants frequently referred to health management for individuals working from home. This has been recommended in Japan to improve infection control since the early phase of the pandemic. Evidence about the effects of working from home on behavioral, health, and economic outcomes is still limited. More evidence, including on how to implement health management, is needed to enable the development of more practical, holistic, and effective occupational health guidelines.

Finally, participants emphasized the importance of the flexible operation of administrative systems at various levels during the pandemic. Similarly, there are many examples of flexibility or resilience in administration in Japan, including research funding, approval for COVID-19 vaccines, public healthcare systems, healthcare at hospitals and clinics, community activities by volunteers, and education at universities. More evidence is needed about what enabled this flexibility and what was more difficult to ensure resilience against future threats to our society.

### Social contribution

The participants confirmed the importance of dialog with society and risk communication between experts and the public during the pandemic. They recognized that experts should disseminate information to respond to inappropriate information circulating on social media and enhance the relationship between medicine and society. This issue is critically important because preventive behaviors among members of the public (e.g., vaccination, mask wearing, physical distancing) affect the pandemic situation. Participants suggested that a population-specific risk-communication approach should be considered (e.g., for foreigners living in Japan), using appropriate channels of communication (e.g., social networking services for younger generations). When communicating with the public, some participants highlighted the importance of using lessons from the history of infectious diseases (e.g., Hansen’s disease), to avoid bioethical problems and violation of any human rights.

To communicate effectively about science, participants perceived the need to ensure that there were suitable opportunities from the preparedness phase. They highlighted the importance of building public trust in experts from the preparedness phase. However, they also perceived difficulties in disseminating expert information. One reason for this may be related to the lack of training in science communication, although the development of resources for risk communication was recommended for universities and academic associations by the Ministry of Education, Culture, Sports, Science, and Technology in Japan after the Great East Japan Earthquake. To our knowledge, only limited opportunities are available for researchers to receive training or education in risk communication in social medicine and public health. Another barrier to risk communication may be related to the assessment system of researchers. One group noted that the weight of outreach activities in the evaluation of researchers’ performance is unclear for early-career researchers, who are often in a precarious employment position. The same problem was also highlighted after the 2011 earthquake. The roles of experts and governments in risk communication were not clearly discussed at the retreat but were a major issue during the early phase of the pandemic in Japan, as well as after the earthquake. Further discussions are needed to clarify the roles of experts and government in risk communication during crises.

### Education

Participants raised issues about online teaching, which was not common before the pandemic but used at most Japanese universities during the pandemic. Communication can help ensure opportunities for education during emergencies, but participants had experienced challenges with online teaching, especially with regard to personal information protection and copyright compliance. This concern was raised from an early phase of this pandemic in Japan. The Ministry of Education, Culture, Sports, Science, and Technology in Japan announced a copyright exemption for education. However, the extent to which this was communicated to teachers might have varied by institution. More practical guidelines on copyright issues from societies or the government may be needed to improve adherence during online teaching.

Another issue is the health impact of online learning. Participants perceived both positive and negative impacts on students’ health and social isolation. At the population level, the causal relationship between online learning and mental health remains unclear, but a recent study in Japan has shown increased suicide rates among undergraduate students during the pandemic. Another Japanese study found that first-year university students in 2020 had higher academic distress than the previous year’s students. The participants suggested that mental healthcare service for students might be an important way to prevent mental health problems and maintain well-being among students.

Third, the participants suggested the importance of cultivating skills specific to online teaching and learning among teachers and students. They also mentioned the difficulty in running practical courses during the pandemic. Experts in medical education have been publishing findings on education during the pandemic in Japan, and collaboration at scale will be important in improving the quality of medical education in Japan.

Finally, participants referred to the disparity in the information environment, including the lack of infrastructure and equipment (e.g., webcam with microphones), as a limitation of online learning. It seems likely that online learning will remain a feature of universities even after the end of the pandemic. Further discussions and solid evidence are needed to develop the “ideal” education using digital technology in medicine.

## Potential Solutions for the Issues

The discussions during the retreat were useful in promoting multidisciplinary communication among young members. One group mentioned that the essence of social medicine does not need to be changed, but its methodologies should be updated to tackle multiple challenges. However, describing perspectives on specific issues is not within the scope of this paper. Therefore, we describe potential solutions based on the JMSF’s activities, especially considering its critical role in creating networks between member societies.

Networking on social medicine may provide some solutions. However, opportunities at the interorganizational or national level are limited. Promoting mutual exchange across academic societies may serve as the basis for developing solutions. Thus, the executive committee is planning the next Social Medicine Young Retreat. In a post-event survey, participants suggested that one solution would be to encourage universities and societies to host academic events to increase knowledge and communication (e.g., seminars, study sessions). Such events would also facilitate consultation and collaboration with appropriate experts by gaining a better understanding from experts and research methods in other disciplines.

Other proposals from participants included starting research groups on specific themes, ideathons (brainstorming events), retreats, research consultations, and mentoring systems. These may also promote research collaboration. These solutions may be feasible and effective because the JMSF can reach many researchers from diverse disciplines in medicine. They may also be acceptable to early-career researchers because many participants expressed expectations about collaboration to address social issues in medicine. The Japanese version of the Centers for Disease Control and Prevention, which the JMSF has suggested establishing to ensure resilience to infectious disease and other health emergencies ^(6)^, will also be a valuable platform to promote multidisciplinary collaboration across societies in social medicine.

The participants have initiated regular meetings for early-career researchers in social medicine and public health, and developed the Social Medicine Young Forum as an official event of the JMSF (Table S3). This action receives administrative support from the JMSF. The aims of this forum are to share early-career researchers’ experiences in research and practice and expand their network across societies in the social division of the JMSF. The forum provides opportunities for early-career researchers/professionals to present their research and practice and exchange opinions. These initiatives could be extended to collaborations with other disciplines, including basic and clinical medicine ^(7)^. Given the complexity of health problems today, collaboration with other fields, including sociology, economics, law, psychology, engineering, and agriculture, are needed in Japan and elsewhere.

This report had some limitations. The participants were not representative of early-career researchers and professionals in social medicine and public health. They included fewer women, students, practitioners, and individuals working in the public or private sectors. The time allotted for discussions was also limited during the retreat, and all possible issues may not have been covered. The situation has changed rapidly during the pandemic, and opinions may also have changed. However, the participants’ perceptions of social medicine’s essential role are unlikely to change substantially. Large-scale surveys among early-career researchers and professionals in social medicine and public health are needed to sort and prioritize issues systematically in a timely manner.

## Conclusion

Early-career researchers who participated in the first Social Medicine Young Retreat in March 2021 described challenges in research, practice, social contribution, and education caused by the COVID-19 pandemic. To tackle these challenges, work is needed to continue and deepen the connections among member societies to increase future collaborative research and encourage other actions. We hope that this article will help to enhance these multidisciplinary exchanges, enabling members to improve lives and health. Organizational and financial support would strengthen the sustainability and scaling-up of these actions.

## Article Information

The opinions presented in this study belong to the participants, which do not necessarily constitute those of the Japanese Medical Science Federation.

### Conflicts of Interest

The authors declare that there are no conflicts of interest. The authors were committee members of the Social Medicine Young Retreat, 2019.

### Acknowledgement

The authors are thankful to Drs Reiko Kishi, Kazuhiko Ohe, Kanae Karita, Chiharu Tohyama, Suminori Akiba, Yuichi Imanaka, and Hiroyasu Iso (JMSF), Dr Hirohito Sone (Japan Epidemiological Association), and participants of the Social Medicine Young Retreat, 2019.

### Author Contributions

Kouji H. Harada substantially contributed to the drafting of the manuscript. Koji Hara and Takuma Yamamoto helped draft the manuscript. Motoki Endo and Mitsuo Uchida helped revise the manuscript. Keisuke Kuwahara made substantial contributions to the conception and design of the work and critically revised it. All authors approved the final manuscript.

### Approval by Institutional Review Board (IRB)

Not applicable.

### Appendix

#### Executive committee members of the retreat

Chair: Keisuke Kuwahara (Japan Epidemiological Association); Vice-Chair: Mitsuo Uchida (Japan Society for Occupational Health); Directors (in charge of Young Retreats): Kanae Karita, Chiharu Toyama (JMSF); Senior Committee Member: Hirohito Sone (Japan Epidemiological Association); Committee Members: Yuka Akiyama (Japan Epidemiological Association), Kaname Iwata (The Japanese Association of Correctional Medicine), Motoki Endo (Japanese Society of Occupational Medicine and Traumatology), Masamitsu Kamada (Japanese Society of Public Health), Shoko Konishi (The Japanese Society of Health and Human Ecology), Tadashi Sawai (Japanese Society for the History of Medicine), Koji Hara (Japan Society for Healthcare Administration), Kouji Harada (Japanese Society for Hygiene), and Takuma Yamamoto (Japanese Society of Legal Medicine).

## Supplement

Supplementary Materials
