# Supplementary material for: Current Issues in Social Medicine and Public Health from the Viewpoint of Early-career Researchers: Summary of Opinions at the Social Medicine Young Retreat
Source: JMA J. 2022 Jun 17;5(3):356–61. doi: 10.31662/jmaj.2021-0219 (PMC9358261; doi:10.31662/jmaj.2021-0219)
Supplement: Supplementary file 1 — Supplementary Materials [file 2433-3298-5-3-0356-s001.pdf]

Appendix Table of Contents

Supplementary text. Additional evidence relevant to issues raised by early career researchers

Figure S1. Top page of special website for Social Medicine Young Retreat 2019

Table S1. Member societies belonging to the social division of the Japanese Medical Science Federation

Table S2. Time schedule of the Social Medicine Young Retreat 2019 held on March 5, 2021

Table S3. Webpages of Social Medicine Young Retreat and Forums

Supplementary text

Scientific evidence relevant to issues raised by early career researchers is provided below as supplementary information.

Research

- The difficulty in data sharing was mentioned during the early phase of the pandemic (e.g., number of COVID-19 cases among healthcare workers) [(1)]. To promote research on the impact of health-related policies, sharing data on policies is important[(2)].
- A Japanese survey showed that 83% of 66 post-doctoral researchers in health sciences perceived the influence of COVID-19 on their research activities in 2020[(3)].
- COVID-19-related restrictions were associated with a decrease in research motivation and anxiety about future research activities, especially among young researchers[(4)].
- Science Council of Japan and Japan Science and Technology Agency, which plays an important role in Japan's Science and Technology Basic Plan, also summarized issues and solutions[(5,6)].

Practice

- In legal medicine, challenges in infection control related to forensic autopsies[(7)]. A report during the COVID-19 pandemic also pointed out the importance of good infection control measures for autopsies[(8)]. Other reports during the COVID-19 pandemic also pointed out the importance of good infection control measures for autopsies[(9,10)].
- While guidelines on implementing practice during the pandemic have been published and updated [(11)], a report described a disparity in implementation of infection control by company size in occupational settings[(12)]. Evidence for implementation of practice is needed[(13)].

- Some practitioners felt hard to find and digest an enormous volume of information on COVID-19 [14].
- A survey in Japan showed that total claimed charges decreased during the early phase of the pandemic[15]. Surveys in Japan showed a 30% drop in the total number of individuals who participated in cancer screening in 2020 and 9.2% drop in the number of people with newly diagnosed cancer between 2019 and 2020 [16,17]. A recent cohort study also found fewer new diagnose of early-stage gastrointestinal cancer during the COVID-19 pandemic [18].
- Japanese studies have shown that income level is not clearly associated with healthcare utilization, diabetes care and telemedicine use [19-21], although these findings may have been influenced by selection bias and timing of exposure assessment.
- Physician shortage in local areas has been a long-standing problem in Japan [22-24] and their working hours will be limited by law in Japan [25]. To maintain the quality of medical services, information and communication technology might be helpful, especially in rural areas [26].
- A recent Japanese survey showed that 54.1% of 37 occupational health practitioners experienced problems when using an online meeting service for interviews[27]. Another Japanese survey found that disparities in telemedicine use had widened across generations during the pandemic[28].
- Working from home or teleworking has been recommended in Japan for infection control since the early phase of the pandemic [11,29]. Evidence on the effect of working from home on behavioral, health, and economic outcomes is limited in Japan [30-35]. Similarly, health management for individuals working from home and effective vaccination procedure in remote settings were raised as challenges [36,37].
- The importance of the flexible operation of administrative systems at various levels during

the pandemic was emphasized by participants. Examples include governmental response to COVID-19[38], research funding[39], approval for COVID-19 vaccines and treatments [40,41], public healthcare systems[42], healthcare at hospitals and clinics[43], community activities by volunteers[44] and education at universities[39].

Social contribution

- Experts should respond to inappropriate information circulating on social media [45]. A recent report in Japan showed that the younger generation was more unsure about and unwilling to have COVID-19 vaccinations than older generations[46].
- Lessons from the history of Hansen's disease are raised in relation to bioethical problems and violation of any human rights [47,48].
- Development of resources for risk communication was recommended by Japanese government [49], while only limited opportunities are available for researchers to receive training or education. Evaluation of researchers' performance in social contribution is unclear for early career researchers, who are often in a precarious employment position [50]. The roles of experts and governments in risk communication were a major issue during the early phase of the pandemic [51].

Education

- Live streaming for remote teaching was used in only 25.5% of universities in Japan in 2016, but used at most Japanese universities during the pandemic[39,52].
- A copyright exemption in teaching was announced by the government [53]. The extent of instruction to teachers about copyright and personal information protection issues might have varied by institution [54-57].
- Problems related to online learning have been pointed out in Japan by researchers in

medical education since the early phase of the pandemic [58,59]. These findings on education during the pandemic will be important in improving the quality [60].

- Increased suicide rates among undergraduate students were observed during the pandemic[61]. First-year university students in 2020 had higher academic distress than the previous year's students [62].
- The disparity in the information environment was identified during the very early phases of the pandemic[63] while online learning has been introduced everywhere. It seems likely that online learning will remain a feature of universities even after the end of the pandemic[64].

References

1. Song PP, Karako T. Scientific solidarity in the face of the COVID-19 pandemic: Researchers, publishers, and medical associations. Glob Health Med. 2020;2:56-9. https://doi.org/10.35772/ghm.2020.01026.
2. Matthay E, Gottlieb LM, Rehkopf D, Tan ML, Vlahov D, Glymour MM. What to Do When Everything Happens at Once: Analytic Approaches to Estimate the Health Effects of Co-Occurring Social Policies. Epidemiol Rev. 2021;43(1):33-47. https://doi.org/10.1093/epirev/mxab005.
3. Saito K, Saito T, Umekawa M, Hoshino T. Survey on the impact of the COVID-19 on research activities-Web Questionnaire Survey on Japan Graduates Database (JGRAD)-. NISTP Res Material, 298, 2022. Available from: https://doi.org/10.15108/rm298. Japanese.
4. Miki Y, Chubachi N, Imamura F, Yaegashi N, Ito K. Impact of COVID-19 restrictions on the research environment and motivation of researchers in Japan. Prog Disaster Sci. 2020;8:100128. https://doi.org/10.1016/j.pdisas.2020.100128.
5. Center for Research and Development Strategy, Japan Science and Technology Agency. A

proposal of building a research platform for infectious disease to make the nation resilient to infectious disease. 2020. Available from: https://www.jst.go.jp/crds/report/CRDS-FY2020-RR-05.html. Japanese.

6. Science Council of Japan. Recommendation: Establishment of a Permanent Organization to Prevent and Control Infectious Diseases. 2020. Available from: https://www.scj.go.jp/ja/info/kohyo/pdf/kohyo-24-t291-4e.pdf. Japanese.

7. Takahama K, Ishizu H, Oshida S, et al., Recommendations for preventing transmission of infection with biohazard in the practice of legal medicine. The Medico-Legal Society of Japan, Committee on Expert Opinions. Nihon Hoigaku Zasshi. 1993;47(2):160-7. https://pubmed.ncbi.nlm.nih.gov/8315871/. Japanese.

8. Nagasaki Y. An autopsy case of COVID-19 in Kobe. Forensic Pathology. 2021;27(1):79-81. Japanese.

9. Tanabe M, Maeda K, Kera H, et al. An autopsy case of detecting SARS-CoV-2 in bodily fluids and organs by real-time RT-PCR. Forensic Pathol. 2021;27(1):75-7. http://houibyouri.kenkyuukai.jp/images/sys/information/20210304053039-C209C5F223A47D28D26CBCC36D591E9B0176CA1B7B4EA334CE68D057E2F50E56.pdf. Japanese.

10. Eberle L, Ihama Y, Graw M. Current situation of COVID-19 at the Institute of Forensic Medicine, Ludwig-Maximilians-University Munich, Germany. Forensic Pathol. 2020;26(1):29-30. Japanese.

11. Japanese Society of Travel and Health, Japan Society for Occupational Health. A guide for measures against COVID-19 at workplaces. Available from: https://www.sanei.or.jp/?mode=view&cid=416. Japanese.

12. Sasaki N, Imamura K, Kataoka M, et al. COVID-19 measurements at the workplace: a two-month follow-up in the cohort study of full-time employees in Japan. Environ Occup

Health Pract. 2021;3(1):eohp.2020-0017-OA. https://doi.org/10.1539/eohp.2020-0017-OA.

13. Brownson R, Fielding JE, Maylahn CM. Evidence-Based Public Health: A Fundamental Concept for Public Health Practice. Annu Rev Public Health. 2009;30:175-201. https://doi.org/10.1146/annurev.publhealth.031308.100134.
14. Ohta R, Matsuzaki Y, Itamochi S. Overcoming the challenge of COVID-19: A grounded theory approach to rural nurses' experiences. J Gen Fam Med. 2021;22(3):134-40. https://doi.org/10.1002/jgf2.410.
15. Shin JH, Takada D, Morishita T, et al., Economic impact of the first wave of the COVID-19 pandemic on acute care hospitals in Japan. PLOS ONE. 2020;15(12):e0244852. https://doi.org/10.1371/journal.pone.0244852.
16. Japan Cancer Society. A 30% decline in cancer screening in 2020, suggesting a possibility of approximately 2100 undiagnosed cases of cancer: A survey by 32 branches in Japan Cancer Society. 2021. Available from: https://www.jcancer.jp/news/11952. Japanese.
17. Japan Cancer Society. Number of diagnosed cases at early stage decreased in 2020, raising a concern of increased cases detected at advanced stage: the first nationwide survey by Japan Cancer Society and three cancer-related associations. 2021. Available from: https://www.jcancer.jp/news/12418. Japanese.
18. Kuzuu K, Misawa N, Ashikari K, et al., Gastrointestinal Cancer Stage at Diagnosis Before and During the COVID-19 Pandemic in Japan. JAMA Netw Open. 2021;4(9):e2126334. https://doi.org/10.1001/jamanetworkopen.2021.26334
19. Aoki T, Matsushima M. The Ecology of Medical Care During the COVID-19 Pandemic in Japan: a Nationwide Survey. J Gen Intern Med 2022. https://doi.org/10.1007/s11606-022-07422-7.
20. Harada M, Nishi T, Maeda T, Tanno K, Nishiya N, Arima H. How do patients with chronic

illnesses respond to a public health crisis? Evidence from diabetic patients in Japan during the COVID-19 pandemic. SSM Popul Health. 2021;16:100961. https://doi.org/10.1016/j.ssmph.2021.100961.

21. Miyawaki A, Tabuchi T, Ong MK, Tsugawa Y. Age and Social Disparities in the Use of Telemedicine During the COVID-19 Pandemic in Japan: Cross-sectional Study. J Med Internet Res. 2021;23(7):e27982. https://doi.org/10.2196/27982
22. Kobayashi Y, Takaki H. Geographic distribution of physicians in Japan. Lancet. 1992;340:1391-3. https://doi.org/10.1016/0140-6736(92)92569-2
23. Tanihara S, Kobayashi Y, Une H, et al. Urbanization and physician maldistribution: a longitudinal study in Japan. BMC Health Serv Res. 2011;11:260. https://doi.org/10.1186/1472-6963-11-260
24. Hara K, Kunisawa S, Sasaki N, et al. Examining changes in the equity of physician distribution in Japan: a specialty-specific longitudinal study. BMJ Open. 2018;8:e018538. https://doi.org/10.1136/bmjopen-2017-018538
25. Tsutsumi A. Workstyle reform for Japanese doctors. Environ Ocup Health Pract. 2020;2(1): eohp.2020-0008-OP. https://doi.org/10.1539/eohp.2020-0008-OP.
26. Sumikawa Y, Yamamoto-Mitani N. Transitional care during COVID-19 pandemic in Japan: Calls for new strategies to integrate traditional approaches with information and communication technologies. Biosci Trends. 2021;15(1):55-7. https://doi.org/10.5582/bst.2021.01056.
27. Morita Y, Ishizawa T, Kajiki S, et al., Occupational health activities under a state of emergency concerning COVID-19 in Japan. Sangyo Eiseigaku Zasshi. 2022;64(1):42-51. https://doi.org/10.1539/sangyoeisei.2020-052-s.
28. Miyawaki A, Tabuchi T, Ong MK, Tsugawa Y. Age and Social Disparities in the Use of Telemedicine During the COVID-19 Pandemic in Japan: Cross-sectional Study. J Med

Internet Res. 2021;23(7):e27982. https://doi.org/10.2196/27982.

29. Ministry of Health, Labour and Welfare. Guideline to promote appropriate introduction and implementation of teleworking. 2021. Available from: https://www.mhlw.go.jp/stf/seisakunitsuite/bunya/koyou_roudou/roudoukijun/shigoto/guideline.html. Japanese.
30. Fukushima N, Machida M, Kikuchi H, et al., Associations of working from home with occupational physical activity and sedentary behavior under the COVID-19 pandemic. J Occup Health. 2021;63(1):e12212. https://doi.org/10.1002/1348-9585.12212.
31. Lunde LK, Fløvik L, Christensen JO, et al., The relationship between telework from home and employee health: a systematic review. BMC Public Health. 2022;22:47. https://doi.org/10.1186/s12889-021-12481-2.
32. Tezuka M, Nagata T, Saeki K, Tsuboi Y, Fukutani N. Association Between Abrupt Change to Teleworking and Physical Symptoms During the Coronavirus Disease 2019 (COVID-19) Emergency Declaration in Japan. J Occup Environ Med. 2022;64(1):1-5. https://doi.org/10.1097/jom.0000000000002367.
33. Shimura A, Yokoi K, Ishibashi Y, Akatsuka Y, Inoue T. Remote Work Decreases Psychological and Physical Stress Responses, but Full-Remote Work Increases Presenteeism. Front Psychol. 2021;12:730969. https://doi.org/10.3389/fpsyg.2021.730969.
34. Nagata T, Nagata M, Ikegami K, et al., Intensity of Home-Based Telework and Work Engagement During the COVID-19 Pandemic. J Occup Environ Med. 2021;63(11):907-12. https://doi.org/10.1097/jom.0000000000002299.
35. Umishio W, Kagi N, Asaoka R, Hayashi M, Sawachi T, Ueno T. Work productivity in the office and at home during the COVID-19 pandemic: A cross-sectional analysis of office workers in Japan. Indoor Air. 2022;32(1):e12913. https://doi.org/10.1111/ina.12913.
36. Nagata T. Impact of increase in working from home. Sangyo Hoken 21. 2021;(105):5-7.

Available from: https://www.johas.go.jp/Portals/0/data0/sanpo/sanpo21/pdf/105.pdf. Japanese.

37. Nakai K. Seeking and considering an efficient COVID-19 vaccination method in remote regions. An Official Journal of the Japan Primary Care Association. 2021;44(4):157-9. https://doi.org/10.14442/generalist.44.157. Japanese.
38. Watanabe T, Yabu T. Japan's voluntary lockdown. PLOS ONE. 2021;16(6):e0252468. https://doi.org/10.1371/journal.pone.0252468.
39. Ministry of Education, Culture, Sports, Science and Technology. Education in Japan beyond the crisis of COVID-19: leave no one behind. 2020. Available from: https://www.mext.go.jp/en/content/20200904_mxt_kouhou01-000008961_1.pdf.
40. Pharmaceuticals and Medical Devices Agency. Special Approval for Emergency on First COVID-19 Vaccine in Japan. 2021. Available from: https://www.pmda.go.jp/english/about-pmda/0002.html.
41. Pharmaceuticals and Medical Devices Agency. Report on special approval for emergency. 2021. Available from: https://www.pmda.go.jp/files/000245005.pdf.
42. Yoshioka-Maeda K. Developing sustainable public health care systems for responding to COVID-19 in Japan. Public Health Nurs. 2021;38(3):470-2. https://doi.org/10.1111/phn.12861.
43. Haruta J, Horiguchi S, Miyachi J, et al., Primary care physicians' narratives on COVID-19 responses in Japan: Professional roles evoked under a pandemic. J Gen Fam Med. 2021;22(6):316-26. https://doi.org/10.1002/jgf2.452
44. Kuroda A, Murayama H, Kurotani K, Fukuda Y, Kuwahara K. A descriptive study on volunteer activities of providing meals during the early phase of the COVID-19 pandemic: clarifying the activities process and a preliminary investigation of the effect of activities on residents. Nihon Koshu Eisei Zasshi, 2022. doi: 10.11236/jph.21-097.

https://doi.org/10.11236/jph.21-097. Japanese.
45. Gallotti R, Valle F, Castaldo N, et al. Assessing the risks of 'infodemics' in response to COVID-19 epidemics. Nat Hum Behav. 2020;4:1285-93. https://doi.org/10.1038/s41562-020-00994-6
46. Yoneoka D, Eguchi A, Nomura S, et al. Identification of optimum combinations of media channels for approaching COVID-19 vaccine unsure and unwilling groups in Japan. Lancet Reg Health West Pacific. 2021;18:100330. https://doi.org/10.1016/j.lanwpc.2021.100330
47. Suzuki N. Manase Dosan (the Elder) and Leprosy. Nihon Ishigaku Zasshi. 1995;41(3):349-68. Available from: http://jsmh.umin.jp/journal/41-3/349-368.pdf. Japanese.
48. Ministry of Health, Labor and Welfare. 2001. The claim for damages by the government against the unconstitutionality of <the Leprosy Prevention Law>. Available from: https://www.mhlw.go.jp/topics/bukyoku/kenkou/hansen/hourei/3.html. Japanese.
49. Committee of Safety, Security, Science, Technology and Social Cooperation, Ministry of Education, Culture, Sports, Science and Technology. Measures to implement risk communication. 2014. Available from: https://www.mext.go.jp/b_menu/shingi/gijyutu/gijyutu2/064/houkoku/__icsFiles/afieldfile/2014/04/25/1347292_1.pdf. Japanese.
50. Jibu M. A study of barriers to the entry into doctoral program. STI Horizon. 2021;7(2):47-52. Available from: https://doi.org/10.15108/stih.00260. Japanese.
51. Sugitani K. Covid-19 as Wicked Problem: Concentrate on the Relation of Politics and Expertise. J Health Welfare Policy. 2021;4(1): 27-37. https://doi.org/10.20690/jhwp.4.1_27. Japanese.
52. Office for University Reform, University Promotion Division, Higher Education Bureau, Ministry of Education, Culture, Sports, Science and Technology. Situation of reform for

educational content at universities in 2016 (Summary). 2019. Available from: https://www.mext.go.jp/a_menu/koutou/daigaku/04052801/__icsFiles/afieldfile/2019/05/28/1417336_001.pdf. Japanese.

53. Fukushima O. 1-2 COVID-19 and Medical Education – Notifications from MEXT and MHLW. Igaku Kyoiku / Medical Education (Japan). 2020;51(3):206-10. Japanese.
54. Kimura T, Suzuki H, Sanuki M, et al., 4-1 Effects of Online Meetings between the Dean and Students on Clerkship at the University of Tsukuba, College of Medicine. Igaku Kyoiku / Medical Education (Japan). 2020;51(3):219-21. https://doi.org/10.11307/mededjapan.51.3_219. Japanese.
55. Ishikawa S, Inoue K, Tomi M. Faculty challenges during the SARS-CoV-2 pandemic and the results of student questionnaires for the implementation of online and face-to-face classes. Jpn J Pharmaceutical Educ. 2021;5:1-6. Available from: https://doi.org/10.24489/jjphe.2020-077. Japanese.
56. Yokoh H, Onodera M, Takahashi Z, et al., 12-1 "Faculty Development to Promote Media Practicum during the Suspension Period of Clinical Clerkship-ICT Support, Giving Curricular Examples and Individual Consultation". Igaku Kyoiku / Medical Education (Japan). 2020;51(3):336-7. https://doi.org/10.11307/mededjapan.51.3_336. Japanese.
57. Iida J. 4-6 Attempt of remote education on anthropology at a university for medical welfare amid the pandemic. Igaku Kyoiku / Medical Education (Japan). 2020; 51(5): 581-3. https://doi.org/10.11307/mededjapan.51.5_581. Japanese.
58. Tanaka M, Abe H. 5-5 Development and Operation of a New Online Education System (F.MOCE) at the University of Fukui Faculty of Medical Sciences, under the COVID-19 Pandemic. Igaku Kyoiku / Medical Education (Japan). 2021;51(3):244-6. https://doi.org/10.11307/mededjapan.51.3_244. Japanese.
59. Nishigori H, Saiki T. 9-6 Consideration for Vulnerable Learners in the Development of

Online Education. Igaku Kyoiku / Medical Education (Japan). 2020;51(3):309-11. https://doi.org/10.11307/mededjapan.51.3_309. Japanese.

60. Takeda Y. Medical Education in the COVID-19 Pandemic. Igaku Kyoiku / Medical Education (Japan). 2021;52(3):185. https://doi.org/10.11307/mededjapan.52.3_185

61. Fuse-Nagase Y, Marutani T, Tachikawa H, et al. Increase in suicide rates among undergraduate students in Japanese national universities during the COVID-19 pandemic. Psychiatry Clin Neurosci. 2021;75(11):351-2. https://doi.org/10.1111/pcn.13293

62. Horita R, Nishio A, Yamamoto M. The effect of remote learning on the mental health of first year university students in Japan. Psychiatry Res. 2021;295:113561. https://doi.org/10.1016/j.psychres.2020.113561

63. Facilities Planning Division, Department of Facilities Planning and Disaster Prevention, Minister's Secretariat, Ministry of Education, Culture, Sports, Science and Technology. A survey and research collaborators conference on planning and enhancement of facilities at national universities for the future (since Reiwa 1 [2019~]) (The 5th). Handout 2-1: Current situations, problems, and future directions. 2020. Available from: https://www.mext.go.jp/b_menu/shingi/chousa/shisetu/056/shiryo/1423025_00005.html. Japanese.

64. Policy Division, Education Policy Bureau, Ministry of Education, Culture, Sports, Science and Technology. 2020 White Paper on Education, Culture, Sports, Science and Technology. 2021. Available from https://www.mext.go.jp/b_menu/hakusho/html/hpab202001/1420041_00009.htm. Japanese.

Figure S1. Top page of special website for Social Medicine Young Retreat 2019. The image is reprinted with permission from The Japanese Medical Science Federation and Senkyo Co., Ltd.

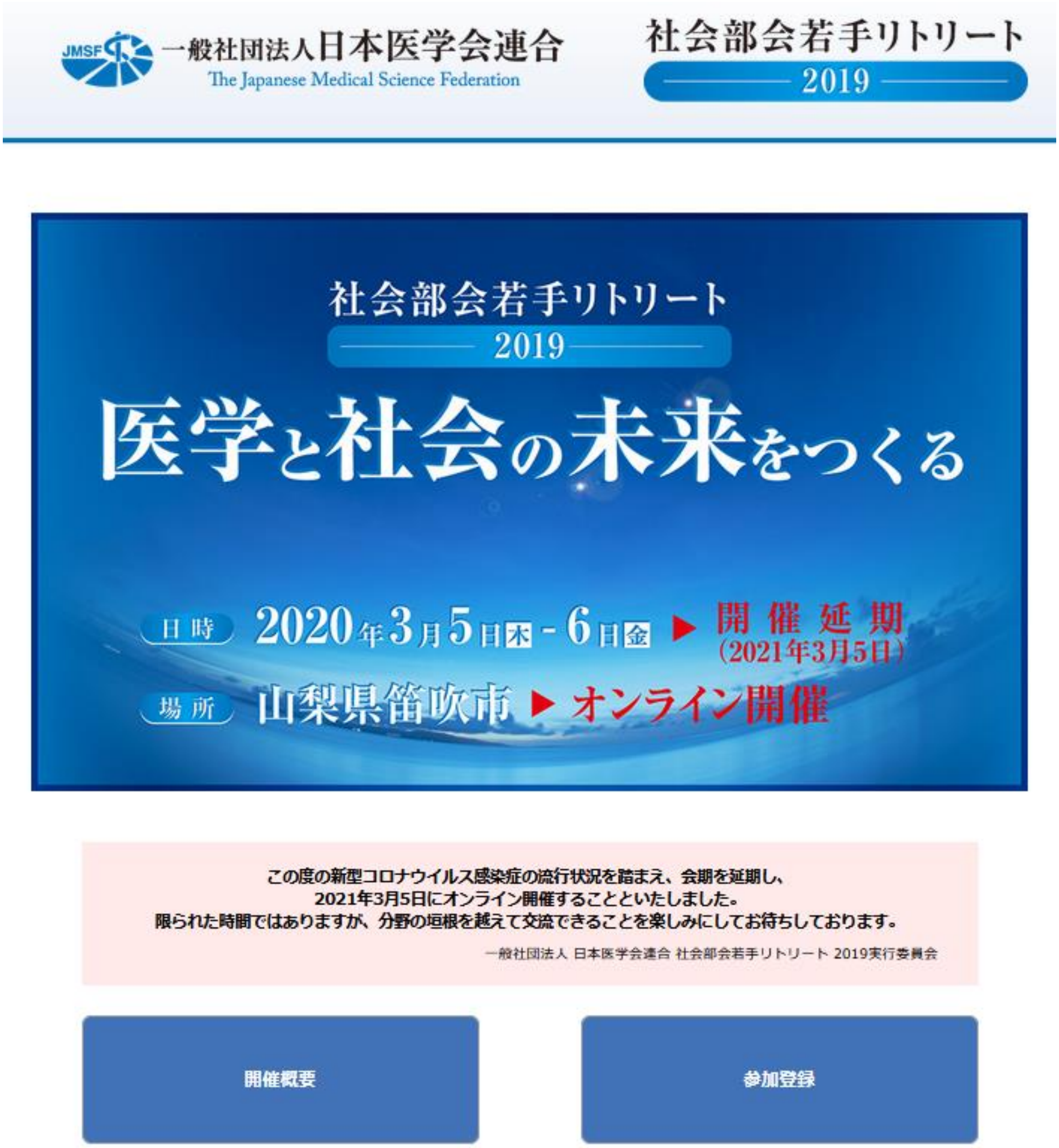

Table S1. Member societies belonging to the social division of the Japanese Medical Science Federation

	Name	Founding year	Website URL
1	Japanese Society of Legal Medicine	1887	http://www.jslm.jp/en/index.html
2	Japanese Society for the History of Medicine	1892	http://jsmh.umin.jp/index_e.html
3	The Association of Insurance Medicine of Japan	1901	https://aimj.org/
4	Japanese Association of Transportation Medicine	1914	http://jatm.umin.jp/
5	Japanese Society of Medical Instrumentation	1923	https://www.jsmi.gr.jp/
6	Japan Society for Occupational Health	1929	https://www.sanei.or.jp/?mode=view&cid=352
7	The Japanese Society for Hygiene	1929	http://www.nihon-eisei.org/en/
8	The Japanese Society of Health and Human Ecology	1930	http://jshhe.com/index.htm
9	The Japan Society of Medical Entomology and Zoology	1943	https://server51.joeswebhosting.net/~js4308/en/
10	Japanese Society of Public Health	1946	https://www.jsph.jp/en/index.html
11	The Japanese Society of Physical Fitness and Sports Medicine	1949	http://www.jspfsm.umin.ne.jp/en/index.htm
12	The Japanese Association of Correctional Medicine	1951	http://jams.med.or.jp/members-s/52.html
13	The Japanese Association of Rural Medicine	1952	http://www.jarm.jp/
14	Japanese Society of Occupational Medicine and Traumatology	1953	http://www.jsomt.jp/
15	Japan Society for Healthcare Administration	1963	http://www.jsha.gr.jp/jsha_en/index.html

16	The Japan Society for Medical Education	1969	http://jsme.umin.ac.jp/eng/index.html
17	Japan Association for Medical Informatics	1983	http://jami.jp/english/
18	Japan Epidemiological Association	1991	https://jeaweb.jp/en/
19	Japanese Association for Disaster Medicine	1995	https://jadm.or.jp/contents/en/

Data were retrieved from https://jams.med.or.jp/members-s/index.html as of Apr 21, 2021

Table S2. Time schedule of the Social Medicine Young Retreat 2019 held on March 5, 2021

Time course	Contents
8:30-9:00	Video introduction of *Schistosoma japonicum*
9:00-9:15	Opening ceremony
9:15-9:25	Self-introduction
**Group work session**	
9:25-10:00	In-group introduction and free-style conversation
10:00-10:50	First group work
10:50-11:00	Break
11:00-11:45	First presentation
11:45-12:05	Second group work
12:05-12:30	Second presentation and feedback from the senior member
**Closing session**	
12:30-12:40	Taking group photos of participants
12:40-12:50	Closing ceremony

Table S3. Webpages of Social Medicine Young Retreat and Forums

Name of the event	Date	Website URL
Social Medicine Young Retreat 2019	Thursday-Friday, March 5-6, 2020 (postponed)<br><br>Friday, March 5, 2021	https://www.jmsf.or.jp/news/page_340.html<br><br>https://www.senkyo.co.jp/sretreat2019/
1st Social Medicine Young Forum	Saturday, June 26, 2021	https://www.jmsf.or.jp/news/page_729.html
2nd Social Medicine Young Forum	Friday, August 27, 2021	https://www.jmsf.or.jp/news/page_938.html
3rd Social Medicine Young Forum	Saturday, December 4, 2021	https://www.jmsf.or.jp/news/page_312.html
4th Social Medicine Young Forum	Friday, March 4, 2022	https://www.jmsf.or.jp/news/page_398.html
